# Criteria for Ending the Distal Fusion at the L3 Vertebra vs. L4 in Surgical Treatment of Adolescent Idiopathic Scoliosis Patients with Lenke Type 3C, 5C, and 6C Curves: Results After Ten Years of Follow-up

**DOI:** 10.7759/cureus.2564

**Published:** 2018-05-01

**Authors:** Mehmet N Erdem, Sinan Karaca, Mehmet F Korkmaz, Meric Enercan, Mehmet Tezer, Ayhan N Kara, Azmi Hamzaoglu

**Affiliations:** 1 Orthopaedics and Traumatology, Hisar Intercontinental Hospital; 2 Orthopaedics and Traumatology, Fatih Sultan Mehmet Education and Research Hospital; 3 Department of Orthopaedics and Traumatology, Inonu University School of Medicine, Malatya; 4 Orthopaedics and Traumatology, Istanbul Florence Nightingale Hospital, Istanbul, TUR; 5 Orthopaedics and Traumatology, Nisantasi Omurga Center

**Keywords:** adolescent idiopathic scoliosis, spinal fusion, surgical treatment, lowest instrumented vertebrae, traction x rays

## Abstract

Introduction

The selection of the most distal caudal vertebra in spinal fusion surgeries in adolescent idiopathic scoliosis patients with structural lumbar curvatures is still a matter of debate. The aim of this study was to determine the preoperative radiological criteria on the traction X-rays under general anesthesia (TrUGA) for selection between the L3 and L4 vertebrae and to assess the efficacy of these criteria via the long-term results of patients with Lenke Type 3C, 5C, and 6C curves.

Methods

Radiological data of 93 patients (84 females, 9 males) who met the inclusion criteria were retrospectively evaluated. The relationship between the L3 vertebra and the central sacral vertebral line, the portion of the L3 vertebra in the stable zone of Harrington, the parallelism of the L3 with the sacrum, and the tilt and rotation of the L3 on TrUGA radiographs were evaluated for the selection of the lowest instrumented vertebrae (LIV). Clinical results were analyzed using the Scoliosis Research Society-22 (SRS-22) questionnaire.

Results

The mean follow-up period of the study group was 149.3 months. According to the Lenke classification, 29 patients had Type 3C, 33 had Type 5C, and 31 had Type 6C curves. The preoperative analysis was based on standing anteroposterior (AP), supine traction, and bending X-rays, and the L3 vertebra was selected as the LIV in 37 patients (40%). These X-rays suggested L4 as the LIV in 56 patients (60%); however, based on our study criteria, the L3 vertebra was selected. No significant loss of correction was observed nor additional surgery due to decompensation was required in the follow-up period.

Conclusion

The use of TrUGA radiographs with the identified criteria is an efficient alternative method in the selection of the LIV in patients with Lenke Type 3C, 5C, and 6C curves.

## Introduction

Preoperative planning of adolescent idiopathic scoliosis (AIS) is a complicated process that requires a series of challenging decisions. The aim is to reduce the amount of deformity and achieve proper fusion that will avoid further progress of the curvature, as well as provide a stable and well-balanced spine. The most critical decision at this point is determining the level of the spinal fusion. Despite the abundant publications on the surgical treatment of idiopathic scoliosis, few have investigated the selection of the fusion site or how the decision is made in structural lumbar curves [1-8].

Ideally, in surgical treatment of scoliosis, the distal end of the fusion should be situated as proximal as possible to preserve the mobile lumbar segments and as distal as possible to avoid trunk imbalance. Therefore, the usual tendency in spine surgeries is to achieve correction through a shorter fusion, leaving as many mobile segments as possible in the distal [2-6,9]. However, the correlation between the length of the fusion and the long-term outcomes is still controversial. In several studies, it has been shown that fusions that extend further than the L3 level cause greater loss of function and higher rates of disc degeneration and more low back pain [10-16]. Few studies reported no difference in terms of function and pain among patients in which the fusion extends to the proximal and distal of the L3 vertebra [17-18]. However, the aim usually is to leave at least three lumbar levels unfused in order to keep as many mobile segments as possible in the lumbar region [2].

In double major or major thoracolumbar/lumbar curvatures, selecting between the L3 and L4 vertebra as the most distal vertebra to be included in the fusion, in other words as the lowest instrumented vertebra (LIV), is usually a challenge for spine surgeons. The aim of this study was to identify the preoperative radiological criteria to end the fusion at the L3 vertebra and to investigate the long-term radiological and clinical data of the patients with Type 3C, 5C, and 6C curves treated with posterior instrumentation and fusion, based on this criteria.

## Materials and methods

Data of the patients who had been diagnosed with AIS and had undergone surgical treatment between January 01, 2002 and January 01, 2008 were retrieved from the files of the Department of Orthopedics and Traumatology at Bilim University and used in the study. The inclusion criteria were as follows: (1) patients who had a treatment of Lenke Type 3C, 5C, and 6C curves with primary posterior instrumentation and fusion; (2) extension of instrumentation to the L3 vertebra at the most distal point; (3) both curves should have been surgically corrected; and (4) a minimum follow-up period of 10 years. Patients who had non-idiopathic scoliosis (e.g. congenital, neuromuscular) or had infantile, juvenile, or adult idiopathic scoliosis, patients who had adolescent idiopathic scoliosis and required surgeries other than posterior spinal fusion, patients with intramedullary pathological findings in the MRI, and those who had disc degeneration that would affect the level of the distal fusion were excluded. Radiological and clinical data of the 93 patients who met the inclusion criteria were retrospectively evaluated.

Standing anteroposterior (AP) and lateral, supine lateral bending and traction radiographs and traction X-rays under general anesthesia (TrUGA) of the patients were taken. The levels for fusion were determined by the senior surgeon (AH). End vertebrae were identified on the standing AP, supine traction, lateral bending, and TrUGA X-rays. Magnitudes of the proximal thoracic, main thoracic, thoracolumbar, and lumbar curves were measured with the Cobb angle. Neutral and stable vertebrae were identified. The curves were grouped according to the Lenke classification. On standing AP, supine traction and TrUGA radiographs, the intersection of the central sacral vertebral line (CSVL) with the vertebrae and whether the CSVL intersected or bisected in particular with the L3 and L4 vertebrae were examined. The L3 and L4 vertebra tilt and L3-L4 disc wedging angle on the coronal plane were measured. The rotation of the L3 and L4 vertebrae was assessed using the Nash and Moe method. The L3 and L4 vertebrae were classified according to the relation with the stable zone of Harrington (SZH). The thoracic kyphosis angle between the T2-T12 and lumbar lordosis angle between the L1-S1 vertebrae was measured on the sagittal plane. Radiological balance was determined by measuring the distance between the CSVL and T1 vertebra on AP radiographs. A trunk shift over 20 mm was accepted as trunk imbalance.

TrUGA radiographs were used for determining the LIV. The following criteria were sought for selecting the L3 vertebra as the LIV: (1) intersection of the L3 vertebra with the CSVL, (2) at least 75% of the L3 fell within the SHZ, (3) L3 vertebra tilt of less than 10 degrees, (4) L3 vertebra parallel/near to parallel the sacrum, (5) a minimum of 1 Nash and Moe grade decrease in the axial rotation of the L3 vertebra when compared to the AP radiographs. In cases that did not meet the above criteria, the fusion was ended at the L4 vertebra as the most distal point.

Bilateral polyaxial pedicle screws were placed along the fusion site and posterior instrumentation and fusion were performed in all patients. All surgical interventions were carried out by the senior surgeon (AH). Surgical outcomes were analyzed by evaluating the stability of the correction, spinal balance, L3 and L4 vertebral tilt, and L3-L4 disc wedging on the standing AP radiographs at the final follow-up. A distance of less than 2 cm between the CSVL and T1 vertebra, an angle of smaller than 10 degrees in the L3 tilt and in the L3-L4 disc wedging, and no increase or an increase of less than 10 degrees in the thoracic or lumbar curves were considered as successful outcomes. Clinical results were analyzed using the Scoliosis Research Society-22 (SRS-22) questionnaire.

The differences between preoperative, postoperative, and follow-up curve magnitudes were analyzed using a repeated measures analysis of variance test. A p value of <0.05 was set for statistical significance.

## Results

Ninety-three patients (84 females, 9 males) were included in the study. The mean follow-up period was 149.3 (range: 120 to 184) months and the mean age of the patients at the time of surgery was 15.2 (range: 13 to 18.4) years. According to the Lenke classification, 29 patients had Type 3C curves, 33 had Type 5C curves, and 31 patients had Type 6C curves (Table 1).

**Table 1:** Demographic data and curve types.

Age (years) Mean ± SD (Median)	15.2 ± 1.4 (15.3)
F/U period (months)	149.3 ± 20.1 (149.5)
Sex	
Male	9
Female	84
Lenke Type	
3C	29
5C	33
6C	31

The relationship of the L3 vertebra within the SZH was assessed on the standing AP and TrUGA X-rays. In 24 cases (26%) 0-25% of the L3 vertebra fell within the SZH on AP radiographs, in 56 cases (60%) 25-50%, and in 13 cases (14%) 50-75% did. On the TrUGA radiographs, 75-100% of the L3 vertebra was included in the SZH in all cases. The L3 vertebra was intersected by the CSVL in 11 cases (12%) on standing AP radiographs. The CSVL did not touch the L3 in the remaining 82 patients (88%). On TrUGA radiographs, the L3 vertebra was bisected by the CSVL in 66 patients (71%) whereas the L3 intersected with the CSVL in 27 patients (29%).

**Figure 1:**
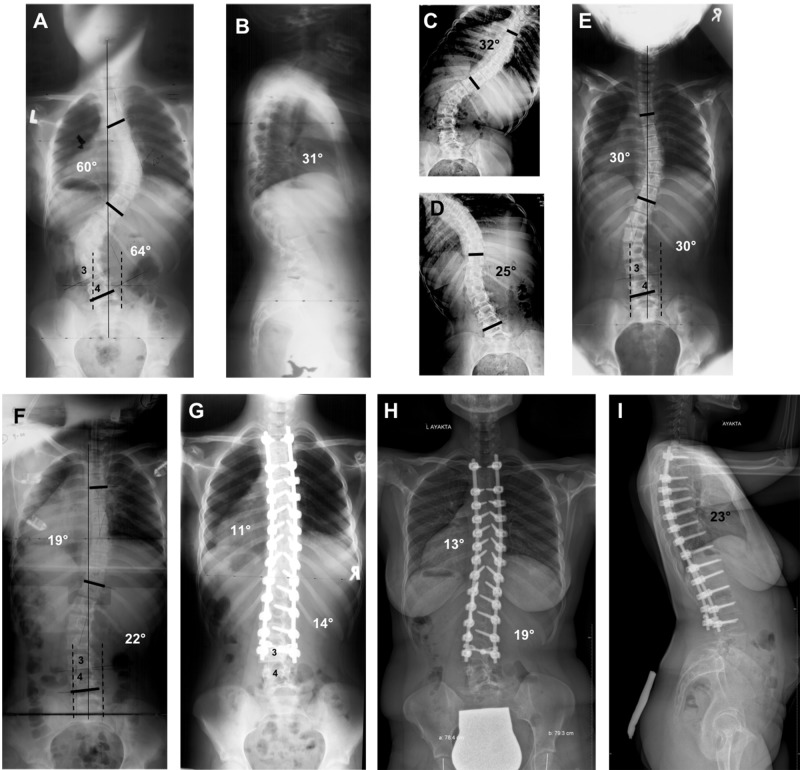
Radiographs of a 13-year-old patient who underwent surgical treatment for Lenke Type 3C adolescent idiopathic scoliosis. (A) Thoracic curvature of 60° and lumbar curvature of 64° are seen on standing AP radiograph. The CSVL comes in contact with the L4 vertebra but not with the L3. 25-50% of the L3 vertebra is in the stable zone of Harrington. (B) Thoracic kyphosis angle of 31° is seen on standing lateral radiograph. (C, D) Bending radiographs of the patient. The CSVL is located toward the lateral of the concave pedicle. If the distal ending point of the fusion is to be decided according to standing AP and bending radiographs, the ideal LIV is the L4 vertebra, not the L3. (E) The CSVL still does not come in contact with the L3 vertebra on the traditional traction radiograph and is located toward the lateral of the concave pedicle. Accordingly, even when the traditional traction radiograph is taken as a reference, the instrumentation is expected to end at the L4 vertebra. (F) On the TrUGA radiograph, the CSVL passes through the medial aspect of the concave pedicle of the L3, 75-100% of the L3 vertebra falls within the SZH, the L3 becomes parallel with the sacrum, the L3 tilt falls below 10° and its axial rotation decreases by 1 grade. Based on these criteria, the L3 could be selected as the LIV, instead of the L4 vertebra. (G) Standing AP radiograph of the patient two years after surgery. (I) Standing AP - lateral radiographs of the patient 14 years after surgery.

**Figure 2:**
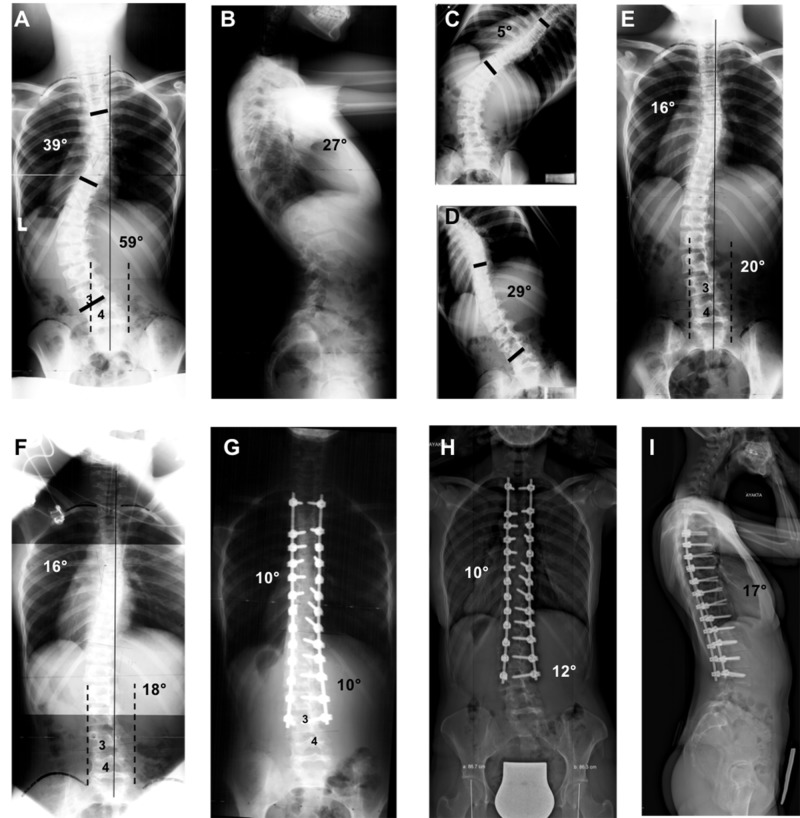
Radiographs of a 16-year-old patient who underwent surgical treatment for Lenke Type 5C adolescent idiopathic scoliosis (AIS). (A) Thoracic curvature of 39° and lumbar curvature of 59° are seen on standing AP radiograph. The CSVL comes in contact with the L4 vertebra but not with the L3. 25-50% of the L3 vertebra is in the stable zone of Harrington. (B) Thoracic kyphosis angle of 27° is seen on standing lateral radiograph. (C, D) Bending radiographs of the patient. The CSVL is located toward the lateral of the concave pedicle on the concave bending radiograph. (E) The CSVL is still located toward the lateral of the concave pedicle of the L3 vertebra on the traditional traction radiograph. (F) On the TrUGA radiograph, the CSVL passes through the medial aspect of the concave pedicle of the L3, 75-100% of the L3 vertebra falls within the SZH, the L3 becomes parallel with the sacrum, the L3 tilt falls below 10° and its axial rotation decreases by 1 grade. Based on these criteria, the L3 could be selected as the LIV, instead of the L4 vertebra. (G) Standing AP radiograph of the patient two years after surgery. (I) Standing AP - lateral radiographs of the patient 12 years after surgery.

The L3 vertebra was selected as the LIV in 37 patients (40%) according to the preoperative analysis using the standing AP, supine traction, and bending radiographs. In 56 patients (60%), the findings from these radiographs suggested that the L4 should be the LIV; however, based on the criteria used in our study, the L3 was selected as the LIV (Figures 1-2).

The mean thoracic curve was 47.1° (range: 31° to 60°), 9.6° (range: 4° to 18°), and 12.8° (range: 4° to 22°) in the preoperative, postoperative, and final follow-up assessments, respectively. The mean lumbar curve was 52.9° (range: 40° to 68°), 11.3° (range: 4° to 17°), and 13.5° (range: 5° to 22°) in the preoperative, postoperative, and final follow-up assessments, respectively. The correction on the coronal plane was preserved during the follow-up, and the difference between the angulation detected in the early postoperative period and final follow-up was found insignificant (p>0.05). The mean thoracic kyphosis angle was 21° (range: 8° to 31°), 27.1° (range: 17° to 34°), and 31° (range: 17° to 38°) in the preoperative, postoperative, and final follow-up assessments, respectively. The same assessments showed the mean lumbar lordosis angle as 32° (range: 20° to 45°), 46° (range: 25° to 62°), and 41.9° (range: 25° to 65°), respectively. No statistically significant difference was detected between the early postoperative and final follow-up sagittal radiological parameters (p>0.05). The distance between the CSVL-T1 vertebra was measured 3.6 (range: 1.2 to 5.4) mm, 0.8 (range: 0 to 1.7) mm, and 0.9 (range: 0 to 1.7) mm in the preoperative, postoperative, and final follow-up assessments, respectively. The L3 vertebra tilt was 24.5° (range: 20° to 32°) preoperatively, 4.4° (range: 0° to 8°) postoperatively, and 4.9° (range: 0° to 8°) at the final follow-up. The tilt of the L4 vertebra was found 20.1° (range: 16° to 26°), 2.2° (range: 0° to 4°), and 3.3° (range: 0° to 6°) at the same time points, respectively. The L3-L4 disc wedging angle was 20.6° (range: 10° to 32°), 4.7° (range: 0° to 12°), and 5.5° (range: 0° to 10°) in the preoperative, postoperative, and final follow-up measurements, respectively. No statistically significant differences were detected between the early postoperative and final follow-up values regarding the CSVL-T1 distance, L3 tilt, L4 tilt, and the L3-L4 disc wedging angle (p>0.05) (Table 2).

**Table 2:** Radiological measurements.

	Preoperative Mean±SD (Median)	Postoperative Mean±SD (Median)	Follow-up Mean±SD (Median)
Main thoracic curve (°)	47.1±7.4 (46)	9.6±4.7 (9)	12.8±5.2 (13)
Thoracolumbar/lumbar curve (°)	52.9±8.3 (50)	11.3±4.8 (12.5)	13.5±5.5 (14.5)
Kyphosis (T2-T12) (°)	21±7.1 (21.5)	27.1±5.2 (28)	31±5.5 (32)
Lordosis (L1-S1) (°)	32±7 (31.5)	46±9.8 (47)	41.9±11.3 (40)
Central sacral vertebral line (mm)	3.6±1.4 (3.9)	0.8±0.5 (0.8)	0.9±0.5 (0.9)
L3 tilt (°)	24.5±3.7 (24)	4.4±1.9 (4)	4.9±2.2 (5)
L4 tilt (°)	20.1±3.3 (19)	2.2±1.1 (2)	3.3±1.8 (3)
L3-L4 disc wedging (°)	20.6±7.5 (22)	4.7±3.7 (4)	5.5±3.2 (5)

None of the patients required additional surgeries due to trunk imbalance, loss of correction, adding-on or decompensation during the follow-up period. The average follow-up SRS-22 score was 4.5 (range: 3 to 5).

## Discussion

The aim of this study was to identify the radiological criteria to end the fusion at the L3 vertebra instead of L4 in the surgical treatment of AIS patients who had Lenke Type 3C, 5C, and 6C curves and to investigate the long-term radiological and clinical data of the operated patients based on this criteria. The results of our study show that in patients where the L4 vertebra is to be selected as the LIV based on standing AP and flexibility radiographs (supine bending and supine traction), it is possible to end the fusion at the L3 through the use of TrUGA X-rays and thus gain one more mobile lumbar segment. Early-term results of this clinical study was published by Hamzaoglu et al. [19]. They investigated Lenke Type 3C and 6C patients. In the current study, patients with Lenke Type 5C curves were also included and long-term outcomes with a minimum follow-up period of 10 years were analyzed.

Advancements in the instrumentation used in the surgical treatment of AIS necessitated several modifications in determining the fusion site. During the times when the Harrington rod was used, selecting the level of fusion was done using standing AP radiographs [1,20,21]. It was concluded that the fusion should take place between the end vertebrae, standing parallel with each other on AP radiographs. Harrington suggested that the most distal vertebra to be included in the fusion, that is the LIV, should be within the SZH [20]. King et al., on the other hand, considered the vertebra bisected by the CSVL as the ‘stable vertebra’ and proposed that the stable vertebra should be included in the fusion in the distal and proximal [1]. If the level of fusion is to be decided according to the stable vertebrae, fusion is ended at the L4 level in the distal, and exceptionally at the L3 level, where instrumentation is supposed to include both the thoracic and lumbar curvatures as is the case in specifically double major or major thoracolumbar/lumbar (Lenke Type 3C, 5C, and 6C) curves.

With the use of pedicle screws, the planning for the level of fusion was begun to be performed using flexibility radiographs such as supine bending or fulcrum bending [2,3,7,8,22-24]. According to the use of bending radiographs in fusion site selection, the fusion should be ended over the neutralized disc gap in the distal on bending radiographs. The same vertebra should also fall within the SZH. Burton et al. termed the ‘caudal foundation vertebra’ (CFV) on the supine side bending radiographs [2]. The CFV was defined as the first vertebra at or above the lower end vertebrae of the lumbar curve that would become centered over the sacrum after the application of torsional reduction loads. Rose and Lenke, on the other hand, defined the LIV according to the CSVL in structural lumbar curvatures and suggested that the lumbar level at the most proximal point intersected by the CSVL on neutral AP radiographs should be the LIV [4].

Beneath the researchers’ efforts in determining the distal fusion level through radiological evaluations lies the aim to attain the highest number of mobile lumbar segments possible following surgical treatment. However, an erroneous LIV selection for this cause may lead to loss of deformity correction, spinal imbalance, and excessive loss of mobile lumbar segments [25]. Selection of the L3 or L4 vertebra as the LIV in thoracolumbar/lumbar curvatures has been a matter of research in several studies. Kim et al. reported successful results with ending the fusion at the L3 level in patients where the midsacral line passes through the L3 vertebra on concave bending radiographs and the rotation falls below Nash and Moe Grade 2 on convex bending radiographs [8]. Wang et al. specified a translation of less than 28 mm and a tilt of less than 25 degrees as the general criteria for LIV selection in patients with Lenke Type 5C scoliosis [25]. Ando et al. defined the preoperative LIV (L3), LIV+1 (L4) translation and L3/4 disc angle on standing, plus LIV+1 translation under traction as important radiological parameters correlating to postoperative global coronal balance in patients who underwent fusion surgery at L3 as the LIV [26]. In another study, Chang et al. reported that in major thoracolumbar/lumbar curvatures, the L3 vertebra should be selected as the LIV if the L3 intersects with the CSVL with a rotation of less than Grade 2 on preoperative bending radiographs; otherwise, the L4 vertebra should be selected as the LIV [27].

Lenke’s classical definition of the LIV was modified by our senior surgeon and redefined as the most proximal vertebra intersected by the CSV on concave bending radiographs but not on neutral AP radiographs. According to this definition, the LIV should be parallel or near parallel with the below vertebra and yet more to the sacrum, and its rotation should decrease by one or two grades on convex bending radiographs. By means of this modification, we were able to end the fusion at the L3 level in many Lenke Type 3C, 5C, and 6C patients instead of the L4 vertebra, which was supposed to be the LIV as it was intersected by the CSVL on neutral AP radiographs. However, even in concave bending radiographs, there are no criteria regarding the distal ending point of the fusion in patients where the L3 vertebra is not intersected by the CSVL. For this reason, TrUGA X-rays, defined by Davis et al. [28] and Hamzaoglu et al. [29] and with its proven efficacy in evaluating the flexibility especially in rigid curves above 65 degrees, had begun to be used in determining between the L3 and L4 vertebra as the ending point of the fusion in double major and major thoracolumbar/lumbar curvatures. The correction rates attained by TrUGA are close to those achieved by posterior instrumentation in Lenke Type 3C, 5C, and 6C curves. Therefore, the LIV was redefined in TrUGA as the vertebra intersected by the CSVL and preoperative criteria were created to enable the selection of the L3 vertebra as the LIV.

Bending and traction and especially TrUGA radiographs in this study showed that the L3 became parallel with the sacrum, that its rotation was decreased by 1-2 grades, and that it was bisected by the CSVL in 37 patients (40%). The decision to end the fusion at the L3 vertebra in the distal can be made without any hesitation in these patients. In 56 patients (60%), the CSVL did not come in contact with the L3 vertebra on standing AP radiographs, the L3 was not parallel with the sacrum on bending radiographs, and it had more than 10 degrees of tilt and its rotation continued on reverse bending radiographs; all pointing out to the L4 as the stable vertebra. On the TrUGA radiographs of these patients, the L3 vertebra was parallel with the sacrum, the CSVL intersected or came in contact with the L3, and more than 75% of the L3 vertebra was included within the SZH. For this reason, based on the data obtained from the TrUGA radiographs of these patients, it was decided to end the fusion at the level of L3 vertebra. None of the 93 patients experienced any decompensation, an increase in the curvature in the fusion site, trunk imbalance, or an angular increase in the L3 tilt or L3-L4 disc wedging during a minimum follow-up period of 10 years (12.5 years on average). These results prove the reliability and efficacy of the criteria for selecting the distal fusion level.

Our study had some limitations. First, the force exerted during the TrUGA radiographs was not standardized. Although all radiographs were taken by the same surgeon and assistant, it was not possible to measure the traction force exerted on each patient. The second limitation was the absence of a control group who did not meet the preoperative criteria and fusion was ended at the L3 level. At this point, we were ethically restricted to form a control group due to the probable increased risk of failure. Finally, the study was planned as a retrospective analysis. Further prospective randomized controlled studies investigating larger patient series are needed.

## Conclusions

In conclusion, the use of TrUGA radiographs is an efficient alternative method in determining the distal fusion level in surgical treatment of patients with Lenke Type 3C, 5C, and 6C curves. Using the criteria determined in TrUGA radiographs, the L3 vertebra was selected as the LIV instead of the L4 and a mobile lumbar segment was spared from fusion in more than half of our patients. In light of these findings, if the CSVL does not come in contact with the L3 vertebra and the L3 does not enter the SZH on standing AP radiographs, and the L3 is not parallel with the sacrum and its rotation continues on reverse bending radiographs in Lenke Type 3C, 5C, and 6C AIS patients; in other words, in TrUGA radiographs where instrumentation is conventionally supposed to be ended at the L4 level in the distal, if: (1) the L3 vertebra is intersected by the CSVL, (2) at least 75% of the L3 vertebra is within the SHZ, (3) the L3 vertebra is parallel with the sacrum, (4) the L3 vertebral tilt is less than 10 degrees, (5) the axial rotation of the L3 vertebra decreases by a minimum of 1 Nash and Moe grade when compared to AP radiographs, then instrumentation can be ended at the level of the L3 vertebra.
